# Lys98 Substitution in Human AP Endonuclease 1 Affects the Kinetic Mechanism of Enzyme Action in Base Excision and Nucleotide Incision Repair Pathways

**DOI:** 10.1371/journal.pone.0024063

**Published:** 2011-09-01

**Authors:** Nadezhda A. Timofeyeva, Vladimir V. Koval, Alexander A. Ishchenko, Murat K. Saparbaev, Olga S. Fedorova

**Affiliations:** 1 Siberian Branch of the Russian Academy of Sciences, Institute of Chemical Biology and Fundamental Medicine, Novosibirsk, Russia; 2 CNRS UMR8200 Université Paris-Sud XI, Institut de Cancérologie Gustave Roussy, Villejuif, France; University of South Florida College of Medicine, United States of America

## Abstract

Human apurinic/apyrimidinic endonuclease 1 (APE1) is a key enzyme in the base excision repair (BER) and nucleotide incision repair (NIR) pathways. We recently analyzed the conformational dynamics and kinetic mechanism of wild-type (wt) protein, in a stopped-flow fluorescence study. In this study, we investigated the mutant enzyme APE1K98A using the same approach. Lys98 was known to hydrogen bond to the carboxyl group of Asp70, a residue implicated in binding the divalent metal ion. Our data suggested that the conformational selection and induced fit occur during the enzyme action. We expanded upon the evidence that APE1 can pre-exist in two conformations. The isomerization of an enzyme-product complex in the BER process and the additional isomerization stage of enzyme-substrate complex in the NIR process were established for APE1K98A. These stages had not been registered for the wtAPE1. We found that the K98A substitution resulted in a 12-fold reduction of catalytic constant of 5′-phosphodiester bond hydrolysis in (3-hydroxytetrahydrofuran-2-yl)methyl phosphate (F, tetrahydrofuran) containing substrate, and in 200-fold reduction in 5,6-dihydrouridine (DHU) containing substrate. Thus, the K98A substitution influenced NIR more than BER. We demonstrated that the K98A mutation influenced the formation of primary unspecific enzyme-substrate complex in a complicated manner, depending on the Mg^2+^ concentration and pH. This mutation obstructed the induced fit of enzyme in the complex with undamaged DNA and F-containing DNA and appreciably decreased the stability of primary complex upon interaction of enzyme with DNA, containing the natural apurinic/apyrimidinic (AP) site. Furthermore, it significantly delayed the activation of the less active form of enzyme during NIR and slowed down the conformational conversion of the complex of enzyme with the cleavage product of DHU-substrate. Our data revealed that APE1 uses the same active site to catalyze the cleavage of DHU- and AP-substrates.

## Introduction

There are a number of DNA lesions that continuously arise in the human genome, resulting in cell death, tumors, autoimmune diseases and aging. It was estimated that about 10^4^ of DNA lesions were generated per mammalian cell per day [1]. The majority of DNA damage can arise either spontaneously or following exposure to reactive oxygen species, derived from a wide range of cellular processes. At present, nearly one hundred different types of damages of heterocyclic bases and sugar-phosphodiester backbone have been identified [2].

Cellular genome integrity is sustained by several distinct repair mechanisms that remove DNA damage. The majority of the damaged DNA bases are eliminated through the base excision repair (BER) pathway [2]. BER is initiated by DNA glycosylases, enzymes that excise damaged and/or mispaired bases to produce apurinic/apyrimidinic sites (AP sites). AP sites also occur through the spontaneous loss of bases (mainly purines) [2]–[4]. AP sites can block DNA replication and transcription and, if bypassed, direct preferential incorporation of A by DNA polymerases [5] that results in single-nucleotide substitution in DNA. During BER DNA is hydrolytically nicked 5′ to the AP site by apurinic/apyrimidinic endonucleases (AP endonucleases). APE1 (35.5 kDa) is the major AP endonuclease in human cells [6], [7].

Repair of certain oxidative base lesions can be initiated directly by the AP endonucleases alone, during nucleotide incision repair (NIR) [8]–[13], by-passing the DNA glycosylase step. During this process, an AP endonuclease introduces a nick 5′ to the damaged deoxynucleotide, generating a 3′-hydroxyl terminus and a 5′-phosphate terminus. The 3′-hydroxyl terminus provides a proper primer for DNA repair synthesis. The dangling damaged nucleotide then can be removed by a flap endonuclease 1 [14]. Thus, the NIR pathway avoids the formation of potentially toxic AP-intermediates [8]. In human cells, NIR is performed by the AP endonuclease APE1 [10]. APE1 was shown to cleave DNA containing 5,6-dihydropyrimidines, α-2′-deoxyadenosine, α-thymidine, α-2′-deoxycytidine, 5-hydroxy-2′-deoxyuridine, and 5-hydroxy-2′-deoxycytidine [10], [12], [13].

It was revealed [15] that the 3′-phosphodiesterase and AP endonuclease activities of the inactive APE1E96A mutant (glutamate-96 was replaced by alanine) were essentially restored to the wild-type protein level in the presence of the second site mutation, K98R (lysine-98 was replaced by arginine). Based on the CD spectra analysis, it was shown that single mutations E96A and K98R or the double mutation E96AK98R did not significantly perturb the protein conformation. However, the APE1K98R and APE1E96AK98R mutants exhibit identical fluorescence emission spectra with lower intensity in comparison to those of the wild-type protein and APE1E96A mutant. The latter two proteins exhibit identical emission spectra. Thus, the K98R mutation has an influence on the structural environment surrounding the tryptophan residues of APE1. Glutamate-96 plays an important role in the co-ordination of the cofactor Mg^2+^. Substitution of glutamate-96 with alanine results in an approximately 400-fold loss of the endonuclease activity [16]–[18]. Restoration of enzymatic activity in the double mutant APE1E96AK98R indicates that the substitution of lysine-98 leads to the formation of an alternative structure. This structure provides efficient Mg^2+^ coordination without involvement of glutamate-96 [15]. Lys98 was shown to take part in the coordination of Mg^2+^, as it hydrogen bonds to the carboxyl group of Asp70 [18]. The latter residue is implicated together with Glu96 in binding the divalent metal ion in the metal binding site, the so-called site A. Besides, the second metal binding site (site B) was found in APE1 at a neutral pH. This site is composed of the side-chains of Asp210, Asn212 and His309. The metal ion in the site A contributes to the stabilization of the transition state intermediate and the 3′-leaving group. The metal in the site B is likely to stabilize the hydroxyl ion that is supposed to act as the nucleophile. This metal is also in a favorable position to neutralize the negative charge of the phosphorane transition-state intermediate [18]–[20]. In addition, there is an assumption that one Mg^2+^ ion moves from the B- to the A-site during substrate cleavage [21].

Many DNA repair enzymes are active versus several structurally different substrates [22]–[23]. Different enzyme conformations are able to recognize these substrates. APE1 also cleaves structurally different substrates. Therefore, the investigation of the influence of different APE1 conformations on the BER and NIR activities is of significant interest.

Recently we proposed [24] that at the initial moment, some enzyme molecules existed in the conformation E1, which was highly active towards DHU-substrate cleavage, whereas a fraction of the enzyme molecules existed in the less active conformation, E2. The less active E2 form of the enzyme can either participate directly in the formation of the initial enzyme–substrate complex, or can be in slow equilibrium with the more active E1 form. Both the complex formation of E2 with the substrate and the conformational transition of E2 into E1 are strongly shifted backward. Furthermore, we demonstrated [24] that APE1 hydrolytically cleaved the DHU-substrate during the NIR pathway and the AP-substrate during the BER pathway with comparable rates.

In the present study, we expand upon the evidence that APE1 can exist in two alternative and distinct conformations. As the mutation of lysine-98 influenced the conformation of APE1, we performed a detailed investigation into how the substitution of lysine-98 with alanine (K98A) affected the enzyme activity in the NIR and BER pathways. To achieve this, we determined the kinetic parameters of the individual stages of APE1K98A action, using the stopped-flow approach. The comparison of the kinetic constants of the mutant to those of wtAPE1 provides us with a view of the mechanistic role of lysine-98. We demonstrate that during both BER and NIR, Lys-98 contributes significantly in the 5′-phosphodiester bond hydrolysis of DNA substrate, but not in the dissociation of the enzyme-product complex. This amino acid substitution influences the catalysis in NIR more than in BER. We also demonstrate the influence of K98A substitution upon the other stages of BER and NIR processes. Based on these data, we suggest that APE1 uses the same active site to catalyze the cleavage of DHU- and AP-substrates. The protein is probably able to form different conformations in the region of the active site, which are responsible for the incision of such structurally unrelated lesions as AP site and DHU.

## Materials and Methods

### Enzyme and oligonucleotides

The mutant APE1K98A protein was expressed in AP endonuclease-deficient *Escherichia coli xth nfo* cells (BH110). *Escherichia coli* strain BH110 (DE3 *nfo::kan^R^* [*Δ(xth-pncA)90 X::Tn10*]) derivative of AB1157 (*IeuB6 thr-1 Δ(gpt-proA2) hisG4 argE3 lacY1 gaIK2 ara-14 mtl-1 xyl-5 thi-1 tsx-33 rpsL31 supE44 r*) (WT) was from the laboratory stock [12]. APE1K98A was constructed and purified as described in [25].

**Figure 1:**
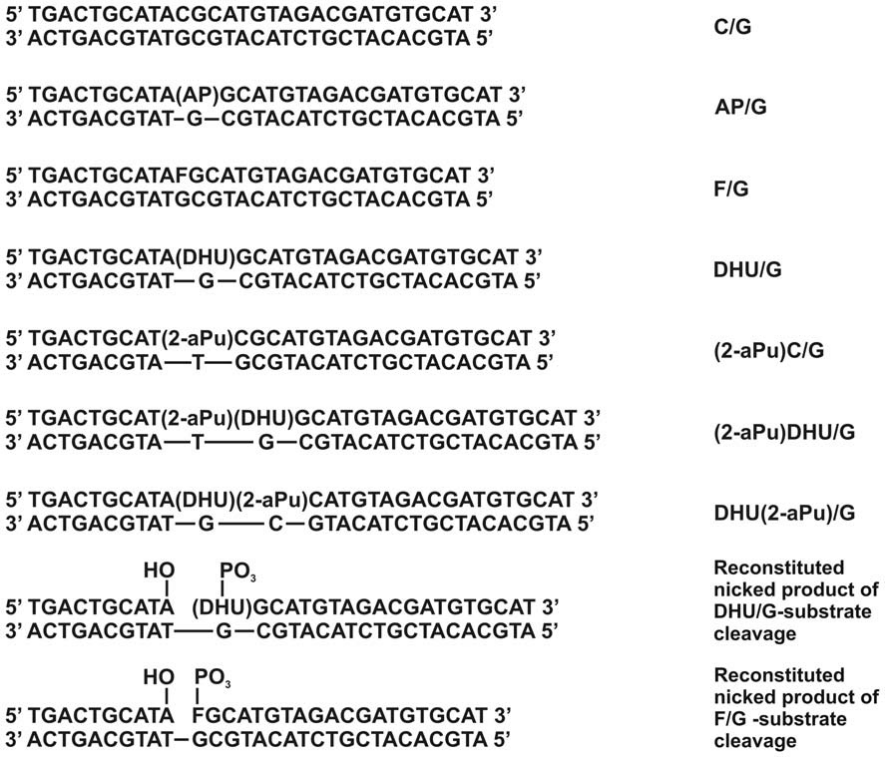
Schematic presentation of various DNA duplexes used in this study.

Oligodeoxyribonucleotides (ODNs) (ATGCACATCGTCTACATGCGTATGCAGTCA), d(TGACTGCATAXGCATGTAGACGATGTGCAT) (X = C, F, U or DHU), d(TGACTGCAT(2-aPu)XGCATGTAGACGATGTGCAT) (X = DHU or C, 2-aPu – 2-aminopurine is a highly fluorescent analogue of adenine), d(TGACTGCATA(DHU)(2-aPu)CATGTAGACGATGTGCAT), d(TGACTGCATA) and pd(XGCATGTAGACGATGTGCAT) (X = F or DHU) were synthesized in the Laboratory of Bionanotechnology (Institute of Chemical Biology and Fundamental Medicine, Siberian Branch of the Russian Academy of Sciences) by the standard phosphoramidite method and purified by an anion exchange and reversed phase HPLC. The concentration of each individual ssODN was ascertained via the absorbance at 260 nm with the extinction coefficients calculated from the nearest-neighbor data for mono- and dinucleotides [26]. In order to generate the AP site d(TGACTGCATAUGCATGTAGACGATGTGCAT) was treated with uracil-DNA glycosylase [27]. When necessary, 5′-termini of the ODNs d(TGACTGCATAXGCATGTAGACGATGTGCAT) (X = F, AP or DHU) were labeled with ^32^P according to the standard procedure [28] using T4 polynucleotide kinase (10–20 U; SibEnzyme, Russia) and [γ-^32^P]ATP (Biosan, Russia). The oligonucleotide duplexes used in this study are presented in Figure 1.

### Stopped-flow kinetics

The kinetics of APE1K98A interactions with DNA substrates was investigated using the stopped-flow method. The kinetic curves were recorded at 25°C with an SX.18MV stopped-flow spectrometer (Applied Photophysics Ltd, Great Britain) detecting the signal of either protein tryptophan residues or substrate 2-aPu residues. Fluorescence excitation and emission maxima of Trp residues in APE1 were observed at λ_ex_ = 281 nm and λ_em_ = 330 nm, respectively [29]. The Trp fluorescence emission of APE1K98A was detected through a long-pass Schott filter WG320 at λ_em_>320 nm with excitation at 281 nm. The 2-aPu fluorescence emission was detected through a long-pass Corion filter LG-370 at λ_em_>370 nm with excitation at 310 nm. The fluorescence was excited using the monochromator equipping the instrument. The dead time of the instrument was 1.37 ms. The signal points were collected at ≥2 ms, except for the case of APE1K98A interaction with the C/G ligand in the BER buffer, where the data at ≥1.5 ms were used. The reaction was carried out in BER (20 mM HEPES/KOH pH 7.5, 50 mM KCl, 5 mM MgCl_2_) and NIR (20 mM HEPES/KOH pH 6.8, 50 mM KCl, 0.01 mM MgCl_2_) buffers. The Trp fluorescence of APE1K98A was recorded for 1 µM concentration of protein in the reaction chamber, and the 0.75–3.0 µM concentration of dsODN substrate. The 2-aPu fluorescence was measured for 1 µM substrate concentration in the reaction chamber, and 0.75–1.5 µM of APE1K98A concentration. During short time periods, each kinetic curve was the average of four or more experiments. For longer times, no averaging was performed. The data were processed using the non-linear regression analysis with the DynaFit software (BioKin, USA) [30]. The kinetic curves were analyzed as previously described [29].

### Steady-state Fluorescence Titration

In order to analyze the affinity of APE1K98A for the reaction product, the enzyme was titrated under steady-state conditions with the ODNs reconstituting the products of DNA cleavage. The Trp fluorescence emission of protein was measured at 330 nm (λ_ex_ = 281 nm) using the Cary Eclipse spectrofluorometer. The excitation and emission wavelengths were adjusted using the monochromators equipping the instrument. Each point of the fluorescence titration curve was obtained by measurement of the fluorescence intensity of separate solutions (80 µl) containing the enzyme (1 µM) and ODN ligand (0.1–7 µM). Each mixture was incubated at 25°C for 10 min before being measured in either the BER or NIR buffer. Fluorescence intensity values (*F*) in the presence of oligonucleotides were corrected for the inner filter effect due to oligonucleotides absorption at 281 nm using the equation:
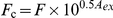
where *F*_c_ is the fluorescence intensity at the particular wavelength, corrected for the inner filter effect, and *A*_ex_ is the absorbance of the oligonucleotides at 281 nm. The dissociation constant (*K*_d_) was calculated based on the dependence of the enzyme fluorescence intensity on the ligand concentration, as described in [29].

### Substrate Cleavage

The time courses of cleavage for 5′-[^32^P]-labeled specific substrates were obtained in the presence of 1 µM APE1K98A and at substrate concentrations of 1, 1.25, and 1.5 µM. The reaction was carried out at 25°C in the BER buffer for F- and AP-containing substrates, and in the NIR buffer for the DHU-containing substrate. The enzyme was rapidly added to the corresponding ^32^P-labeled substrate. The reaction was quenched at designated intervals by the addition of reaction mixture aliquots into a solution containing 7 M urea with marker dyes. The nicked products were separated in 20% denaturing PAGE. The gels were dried, visualized using a Molecular Imager FX phosphorimager (Bio-Rad, USA), and quantified using the Gel-Pro Analyzer software (Media Cybernetics, USA). The data obtained for DHU-substrate were analyzed with the DynaFit software (BioKin, USA) [30].

## Results

### Interaction of APE1K98A with undamaged ligand in BER and NIR buffers

**Figure 2:**
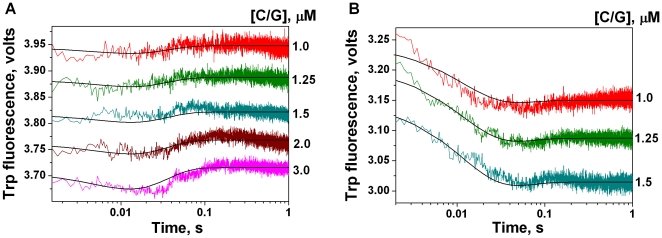
Trp fluorescence traces of APE1K98A (1 µM) upon binding with the C/G ligand. The kinetic curves were recorded in the BER (**A**) and NIR (**B**) buffers. Smooth curves are a result of the fitting procedure. Concentrations of C/G are indicated next to the right axis.

**Figure 3:**
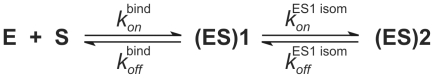
The kinetic scheme describing the interactions of APE1K98A with undamaged ligand.

The interaction of APE1K98A with the undamaged 30-mer DNA ligand (C/G) resulted only in the formation of the enzyme-substrate complex. Initially, a decrease in the protein fluorescence intensity occurred in the BER buffer during the time range of 0–10 ms (Figure 2A), and in the NIR buffer during the time range of 0–40 ms (Figure 2B). The fluorescence intensity then showed an increase, and the traces entered a plateau phase at times exceeding 10–20 ms in the BER buffer, and 50 ms in the NIR buffer. The decrease likely reflected the formation of an initial non-specific complex. It was supposed that the following increase in the fluorescence corresponded to the isomerization of the initial complex to yield the second protein-DNA complex. During this process the protein underwent conformational adjustment. Such double-stage binding in both BER and NIR buffers was described by the kinetic scheme presented in Figure 3. Processing of the kinetic curves according to this scheme provided the rate constants values of APE1K98A interaction with the C/G ligand (Table 1).

**Table 1:** Rate constants of APE1K98A and APE1 binding with undamaged C/G ligand.

	APE1K98A		APE1(a)	
Constants	BER buffer	NIR buffer	BER buffer	NIR buffer
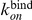 (M^−1^ s^−1^)	(2.0±0.1)×10^6^	(3.8±0.1)×10^6^	1.1×10^7^	4.4×10^5^
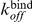 (s^−1^)	78±2	71±1	200	12
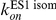 (s^−1^)	8.3±0.1	0.23±0.01	16	0.82
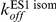 (s^−1^)	59±1	28±0.5	80	1.5

a Data taken from [29].

### Interaction of APE1K98A with F- and AP-substrates in BER buffer

Except for the natural AP site, human AP-endonuclease can recognize and process the synthetic analog of deoxyribose, (3-hydroxytetrahydrofuran-2-yl) methyl phosphate (F). Herein, we investigated the conformational dynamics of APE1K98A interaction with the substrates containing both the natural AP site and the F-residue in the BER buffer.

Experiments with [^32^P]-labeled substrates AP/G and F/G analyzing the product accumulation revealed that at the used concentrations, the enzyme cleaved these substrates completely at the first time point sampled (∼10 s, data not shown).

**Figure 4:**
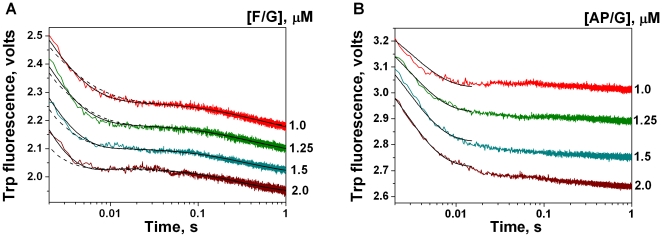
Trp fluorescence traces of APE1K98A (1 µM) interacting with substrates F/G (A) and AP/G (B). The reactions were carried out in BER buffer. Smooth curves are the result of the fitting procedure. Traces were fitted to kinetic schemes containing a single (A, dashed curves) or a double (A, B, solid curves) binding equilibrium. Concentrations of the substrates are indicated next to the right axis.

Upon interaction of the enzyme with both specific substrates the changes in fluorescence intensities on the kinetic curves were stronger than in the case of the C/G ligand (Figure 4).

In the case of the substrate F/G the fluorescence intensity was decreasing for ∼10–20 ms. The fluorescence curves then underwent a kink at around 0.1 s, which was followed by a slow decrease in fluorescence. The fluorescence traces were fit to kinetic schemes containing a single or a double binding equilibrium (Figure 4A). The double-equilibrium binding scheme matches the data better. Values of standard deviations for the double-equilibrium binding scheme were lower than that for the single-equilibrium binding scheme. These results suggested that the formation of the initial enzyme-substrate complex was followed by the isomerization of this complex.

**Figure 5:**
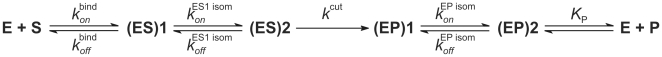
The kinetic scheme describing the interactions of APE1K98A with specific F- and AP-substrates during BER.

Based on the data obtained we have proposed the kinetic scheme describing the mechanism of the F-substrate conversion by APE1K98A in the BER pathway (Figure 5). This scheme includes two reversible steps: initial complex formation and isomerization, followed by an irreversible step of 5′-phosphodiester bond hydrolysis, a reversible step of enzyme-product complex isomerization, and a reversible step of enzyme dissociation from the complex with the product. Figure 4A clearly illustrates that fluorescence intensities do not return to their initial values. This indicates that the terminal dissociation step is not reflected on the fluorescence kinetic curves. Thus, the enzyme-product complex is rather stable. The rate constants values of APE1K98A interaction with specific substrate F/G are presented in Table 2.

**Table 2:** Rate constants of APE1K98A and APE1 interactions with specific F- and AP-substrates in BER buffer.

	APE1K98A		APE1(a)	
Constants	F/G	AP/G	F/G	AP/G
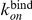 (M^−1^ s^−1^)	(3.7±0.1)×10^8^	(2.0±0.1)×10^8^	3.8×10^8^	1.8×10^8^
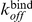 (s^−1^)	38±1	492±2	79	120
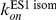 (s^−1^)	30±1	289±1	59	18
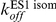 (s^−1^)	310±1	355±2	18	32
*k* ^cut^ (s^−1^)	5.5±0.1	N/D	68	97
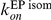 (s^−1^)	2.7±0.1	N/D	N/D	N/D
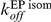 (s^−1^)	1.5±0.1	N/D	N/D	N/D

a Data taken from [29].

The APE1K98A reaction with the substrate AP/G was accompanied by a decrease in the fluorescence intensity for ∼10–30 ms (Figure 4B). The subsequent changes in fluorescence intensity were less pronounced than that for the substrate F/G. The kinetic traces of enzyme interaction with AP/G allowed us to fit only the rate constants of the first two reversible steps corresponding to the initial complex formation and its isomerization (Table 2). Thus only the initial decrease in the fluorescence intensity up to 15 ms was quantitatively processed. At the same time the shapes of kinetic curves for both specific substrates were similar. According to these data we assumed that the number of kinetic stages during the enzyme interaction with the substrates AP/G and F/G was equal (Figure 5).

The fluorescence titration of protein with reaction product was performed under steady-state conditions, in order to determine the equilibrium dissociation constant for the complex of APE1K98A with the product (*K*_P_). The reaction product containing an AP site at the 5′-terminus at the site of the nick was very unstable [31]. Therefore, three oligonucleotides were used to reconstitute a reaction product with an F-residue at the 5′-terminus at the site of the nick. Such a complex was stable at the reaction temperature (25°C) [29] (see Materials and Methods). APE1K98A was titrated with this complex and changes in the Trp fluorescence intensity were recorded (data not shown). The resulting *K*_P_ value was 0.44±0.1 µM.

### Interaction of APE1K98A with DHU-substrate in NIR buffer

5,6-Dihydropyrimidines are major DNA base lesions generated by γ-irradiation under anoxic conditions [32]. They are substrates for the AP endonucleases-catalyzed NIR activity. Therefore, we investigated the APE1K98A interaction with the DHU-containing substrate.

### APE1K98A cleavage of [^32^P]-labeled DHU-substrate

**Figure 6:**
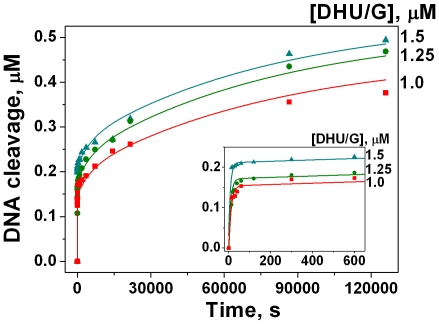
Time course of [^32^P]-DHU/G substrate cleavage by APE1K98A (1 µM). The reaction was carried out in NIR buffer. Concentrations of the substrate are indicated next to the right axis. The initial region of the kinetic curves is shown on the insert that corresponds to the phase 1 observed in the time range <100 s. Phase 2 is observed in the time range 100<t<20000 s. Phase 3 corresponds to the slow accumulation of cleaved DNA at t>20000 s.

We obtained the time course of accumulation of incised product, during processing of 5′-[^32^P]-labeled DHU-substrate by APE1K98A in NIR buffer (Figure 6). Mutant protein was found to cleave about 35% of substrate during 35 hours (Figure 6), whereas APE1 cleaved slightly more than 60% of substrate during the same amount of time [24]. Apparently, mutant protein form operated more slowly than the wild-type protein.

Three phases could be recognized on the kinetic curves of product accumulation (Figure 6). Within the first phase in the time range <100 s the rapid product accumulation with reaching an intermediate plateau was observed. During the next two phases the products accumulated more slowly. The concentration of products generated during the first phase of the process was lower than the initial concentration of the enzyme (Figure 6). It amounted to ∼15% of the initial substrate concentration. The same result was observed in the case of the wild-type protein [24]. In all likelihood, the mutant form APE1K98A behaves like the wild-type protein when binding the DHU-substrate. The decreased amplitude of initial product accumulation suggests that the enzyme exists in the equilibrium at least between two conformations.

The second phase of product accumulation was observed at 100<t<20000 s. The third phase corresponded to an extremely slow accumulation of the product and continued at t>20000 s. The slow biphasic accumulation of the product makes a strong case for a reaction with a less active form of the enzyme and for an existence of the stable enzyme-product complex (Figure 6).

### Kinetic traces of 2-aPu fluorescence of DHU-substrates upon the interaction with APE1K98A

**Figure 7:**
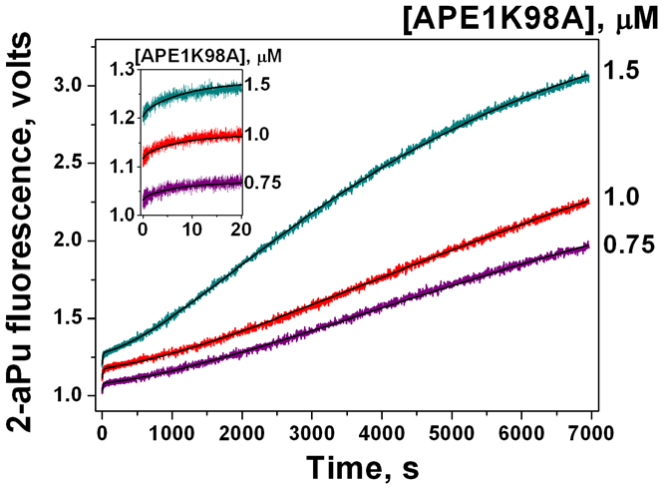
2-aPu fluorescence traces of the substrate (2-aPu)DHU/G (1 µM) upon interaction with APE1K98A. The reaction was carried out in NIR buffer. Smooth black curves are the result of the fitting procedure. Concentrations of the enzyme are indicated next to the right axis. The insert shows the initial region (<20 s) of the fluorescence intensity increase, reaching an intermediate plateau. This region corresponds to phase 1. Phase 2 is a subsequent increase in the fluorescence intensity up to ∼4000 s. The increase in the fluorescence intensity at >4000 s corresponds to the beginning of phase 3.

We examined the changes in fluorescence intensity of a 2-aPu residue located in the substrate at the 5′-side of DHU in the presence of enzyme in NIR buffer, in order to determine the kinetic mechanism of APE1K98A interaction with DHU-substrate. Although no changes were observed in the 2-aPu fluorescence intensity upon APE1K98A binding with undamaged DNA ((2-aPu)C/G) (data not shown), the changes of 2-aPu fluorescence intensity were observed with (2-aPu)DHU/G substrate. The shapes of kinetic curves were the same as in the case of the wild-type protein [24], however the processes occurred more slowly (Figure 7). It was possible to differentiate between three phases on these curves. Within the initial rapid phase (<20 s (Figure 7)) the 2-aPu fluorescence intensity increased, reaching an intermediate plateau. This initial burst of the fluorescence intensity coincided with the phase of the initial accumulation of product of [^32^P]-DHU/G substrate cleavage (Figure 6, Phase 1). The second phase of the fluorescence intensity increase was observed up to ∼4000 s. The beginning of the third phase was recorded at >4000 s. (Figure 7). The latter phase corresponded to the third phase of the slow accumulation of product of [^32^P]-DHU/G substrate cleavage (Figure 6).

In addition, we examined the APE1K98A interaction with the substrate DHU(2-aPu)/G, which contained 2-aPu residue located at the 3′-side of DHU, in NIR buffer. No significant changes of 2-aPu fluorescence intensity were registered up to 7000 s (data not shown).

### Kinetic changes of Trp fluorescence intensity upon the interaction of APE1K98A with DHU-substrate

**Figure 8:**
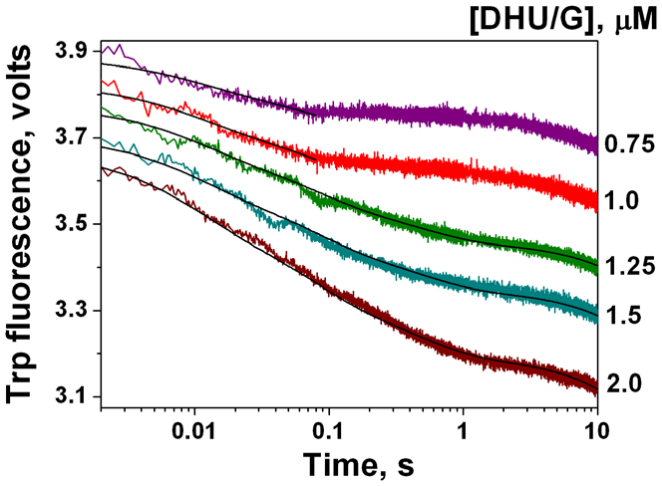
Trp fluorescence traces of APE1K98A (1 µM) upon interaction with the substrate DHU/G. The reaction was carried out in NIR buffer. Smooth curves are the result of the fitting procedure. Concentrations of the substrate are indicated next to the right axis.

The conformational changes of APE1K98A upon its interaction with the substrate DHU/G were measured by the tryptophan fluorescence of protein in NIR buffer. As shown in Figure 8 the fluorescence intensity sharply decreased up to 0.1 s with substrate concentrations 0.75 µM, 1 µM, and up to 1 s with substrate concentrations 1.25 µM, 1.5 µM, 2 µM. Then the fluorescence signal decreased more slowly. Thus for substrate concentrations ≥1.25 µM it was possible to observe the additional stage, absent in the case of lower substrate concentrations. If the time exceeded 10 s, the protein bleaching became noticeable. This fact was observed during the registration of protein fluorescence traces in the absence of substrate (data not shown). The shapes of kinetic curves after the correction of bleaching became ambiguous. Therefore, we registered the Trp fluorescence only up to 10 s.

The decrease of protein fluorescence intensity in the time range ≥1 s (Figure 8) was consistent with the initial increase in (2-aPu)DHU/G fluorescence intensity (Figure 7, Phase 1). Hence, this decrease corresponded to the first rapid phase of substrate cleavage. We suggest that the mutant protein exists in two conformations, as in the case of wtAPE1 [24]. At the initial moment one part of the enzyme exists in the conformation E1, which is energetically less advantageous but more active for DHU-substrate cleavage. Another part of the enzyme exists in the conformation E2, which is energetically more advantageous but significantly less active for the cleavage of this substrate. Therefore the initial sharp decrease of protein fluorescence intensity (Figure 8) is likely to correspond to the substrate binding by the E1 form of enzyme, and to the subsequent isomerization of this complex.

### Kinetic scheme of APE1K98A action in the nucleotide incision repair pathway

**Figure 9:**
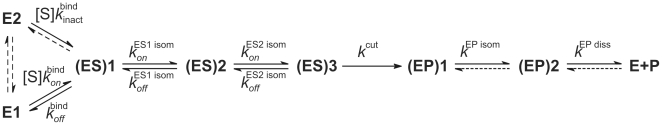
The kinetic scheme describing the interactions of APE1K98A with specific DHU-substrate during NIR.

Analysis of Trp fluorescence traces of APE1K98A interacting with DHU/G (Figure 8) reveals the existence of four stages corresponding to the initial complex formation of E1 with the substrate, two stages of complex isomerization, and the stage of chemical cleavage of substrate (Figure 9). The forward and reverse rate constants of formation of initial enzyme-substrate complex (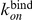, 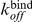), and of this complex isomerization (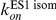, 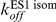, 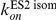, 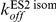), as well as the rate constant of substrate cleavage (*k* ^cut^) (Figure 9) are presented in Table 3. Only the initial steps corresponding to the first two equilibrium stages can be quantitatively processed on kinetic curves, obtained for low DHU/G concentrations 0.75 µM and 1 µM.

**Table 3:** Rate constants of APE1K98A and APE1 interactions with DHU-containing substrates in NIR buffer.

	APE1K98A			APE1(a)		
Constants	DHU/G (from Fig. 8)	[^32^P]-DHU/G (from Fig. 6)(b)	(2-aPu)DHU/G (from Fig. 7)(b)	[^32^P]-DHU/G	(2-aPu)DHU/G	DHU(2-aPu)/G
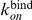 (M^−1^ s^−1^)	(9.2±0.1)×10^6^					
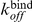 (s^−1^)	81±1					
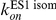 (s^−1^)	4.1±0.1					
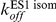 (s^−1^)	13±0.4					
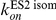 (s^−1^)	1.7±0.1					
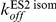 (s^−1^)	2.2±0.1					
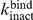 (s^−1^)		(1.9±0.6)×10^−4^		1.6×10^−2^		
*k* ^cut^ (s^−1^)	0.20±0.01	0.22±0.03	0.30±0.01		49	53
*k* ^EP isom^ (s^−1^)			(4.4±0.7)×10^−4^		3.7×10^−3^	2.7×10^−3^
*k* ^EP diss^ (s^−1^)(c)		(1.2±0.5)×10^−5^		1.3×10^−5^	1.5×10^−5^	

a Data taken from [24].

b The amount of enzyme involved in the substrate cleavage was 15% of the initial APE1K98A concentration.

c The error in *k* ^EP diss^ includes the contribution of APE1 inactivation as a result of long-term incubation.

The reaction progress curves for cleavage of [^32^P]-DHU-substrate by APE1K98A (Figure 6) illustrate the three phase behavior. Based on the proposed kinetic scheme (Figure 9) it can be supposed that the first burst phase corresponds to the activity of enzyme existing in the more active conformation E1 (Figure 6, Phase 1). The slower second stage, as in the case of the wild-type protein, is limited either by the rate of the direct enzyme-substrate complex formation of the less active enzyme form E2 or by the rate of conformational transition of E2 into E1 (Figure 6, Phase 2). The third, slowest stage can be attributed to the process of APE1K98A release from the complex with the nicked product (Figure 6, Phase 3). The latter stage seems to be limiting the overall enzymatic process and determining its rate in the steady-state conditions. The rate constants of the first (*k* ^cut^), second (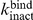), and third (*k* ^EP diss^) phases are presented in Table 3.

2-aPu fluorescence traces recorded for (2-aPu)DHU/G substrate upon interaction with APE1K98A (Figure 7) also demonstrate the three phase character. Apparently, the first phase corresponds to the rapid chemical substrate cleavage by the enzyme. As in the case of wild-type protein, we suggest that the second phase in Figure 7 is limited by a slow conformational change of the APE1K98A complex with the nicked product. Hence, this second phase does not correspond to the process limiting the second stage of accumulation of the [^32^P]-labeled substrate nicked product (Figure 6). The rate constants corresponding to the first (*k* ^cut^) and second (*k* ^EP isom^) phases differentiated in Figure 7 are presented in Table 3. One can see that the second phase of slow 2-aPu fluorescence intensity increase (Figure 7) is limited by the more rapid process (*k* ^EP isom^) than the second phase in Figure 6 (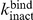). The third phase (Figure 7) was ascribed to the enzyme release from the stable complex with the product. Due to the fact that only the beginning of process was observed on kinetic curves, there was no opportunity to quantitatively process the latter phase.

The values of rate constants of chemical cleavage (*k* ^cut^) obtained from the kinetic curves of APE1K98A interaction with substrates DHU/G (Trp fluorescence detection), (2-aPu)DHU/G (2-aPu fluorescence detection), and [^32^P]-DHU/G (detection of product accumulation) showed a high degree of similarity (Table 3). The rate constant of limiting stage corresponding to release of APE1K98A from the complex with the product (*k* ^EP diss^ = 1.2×10^−5^ s^−1^, Table 3) coincides with the appropriate value for APE1 (*k* ^EP diss^ = 1.3×10^−5^ s^−1^) [24].

The equilibrium constant for dissociation of APE1K98A complex with the product *K*_P_ was determined by the fluorescence titration with the reaction product under steady-state conditions, as described earlier (data not shown). To reconstitute the reaction product we used the nicked oligonucleotide duplex containing the DHU residue at the 5′-terminus at the site of the nick. The fitting value of *K*_P_ is equal to 1.9±0.2 µM.

## Discussion

To incise the DNA sugar-phosphate backbone at 5′ to such structurally unrelated lesions as AP site and DHU, it is necessary that APE1 should undergo the conformational change of its active site region. In accordance with the concept of conformational selection [33], [34], all proteins pre-exist in a dynamic equilibrium between various conformations. Multiple-ligand binding at a single site simply reflects the existence of the distribution of conformational isomers of enzyme in solution. The ligand defines the binding site shape and size. Different ligands selectively bind to different conformers. The range of dissimilar ligands that will bind is dependent upon the distribution and redistribution of the conformations. Mutations in protein are likely to lead to population shifts in pre-existing conformers. It is known, that Lys98 takes part in coordination of Mg^2+^, as it hydrogen bonds to the carboxyl group of Asp70, a residue implicated in binding the divalent metal ion in the metal binding site A [18]. The metal ion in this site was shown to play a role in stabilizing the transition state intermediate and the 3′-leaving group [18]–[20]. Furthermore, the substitution of lysine-98 leads to the formation of an alternative structure [15] near the active site. It should influence the kinetic behavior of the enzyme. In the present study, the pre-steady-state kinetic analysis of APE1K98A mutant reveals a detailed view of the role of lysine-98 in the enzymatic catalysis. Our data implies that the conformational selection does take place upon the protein binding of DHU-substrate and that the enzyme pre-exists in the equilibrium at least between two conformations. What is more, the enzyme in the complex with ligands undergoes the conformational changes, indicating that the induced fit pathway takes place in addition to conformational selection.

### APE1K98A interaction with undamaged ligand

The interactions of APE1K98A with undamaged ligand C/G in both BER and NIR buffers are described by the kinetic scheme presented in Figure 3. This scheme includes two reversible equilibrium stages: formation of the initial enzyme-substrate complex and the isomerization of this complex. In different buffers the corresponding rate constants of the individual reaction steps are varied, illuminating the effect of pH and Mg^2+^ on the enzyme activity. In the NIR buffer the forward rate constant of initial enzyme-substrate binding (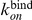) is approximately twofold higher than in the BER (Table 1). At the same time the reverse rate constants of the first equilibrium (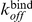) are almost the same in two different buffers. For APE1 (Table 1, [29]) the forward rate constant of initial unspecific complex formation (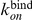) in BER buffer exceeded the same constant in the NIR buffer by more than an order of magnitude. At the same time, the reverse rate constant for this step (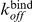) was also more than 10-fold higher in BER buffer, than in NIR. In BER buffer the formation rate of unspecific complex (ES)1 of APE1 is 5-fold higher and the stability of this complex (calculated as 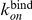/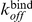) is twofold higher than in the case of APE1K98A. At the same time in NIR buffer the formation rate and the stability of complex (ES)1 are higher for APE1K98A than for APE1 9-fold and 1.5-fold, respectively.

Isomerization of the complex of APE1K98A with an unspecific substrate occurs faster in BER buffer than in NIR. In BER buffer the complex (ES)2 is 17-fold more stable than in NIR (the stability is calculated as 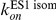/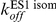). In the case of the wild-type protein (Table 1, [29]) the rate of isomerization was also greater in BER buffer than in NIR buffer, and in both buffers it was found the isomerization occurred faster than in the case of APE1K98A. The stability of (ES)2 of APE1 exceeds the stability of the APE1K98A complex in both BER (1.4-fold) and NIR (67-fold) buffers.

Hence, the substitution of lysine-98 in APE1 by alanine causes the formation rate and stability of the initial unspecific enzyme-substrate complex (ES)1 to increase in NIR buffer and decrease in BER buffer. At the same time, the isomerization rate of the initial complex and the stability of the complex (ES)2 decrease in both buffers used. What is more, the stability of complex (ES)2 in NIR buffer decreases significantly (67-fold).

The data obtained in this work indicate that the mutation K98A influences the formation of the primary unspecific enzyme-substrate complex in a complicated manner, depending on the Mg^2+^ concentration and pH. This mutation almost eliminates the significant differences observed for APE1 between corresponding constants 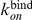 and 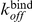 in two buffers used. What is more, the substitution of Lys-98 by Ala obstructs the induced fit of the enzyme in the complex with undamaged DNA.

### APE1K98A interaction with specific F- and AP-substrates in BER buffer

In the case of APE1K98A interactions with specific F- and AP-substrates two reversible steps (initial enzyme-substrate complex formation and its isomerization) are followed by an irreversible chemical step of substrate 5′-phosphodiester bond hydrolysis, a reversible step of enzyme-product complex isomerization, and a reversible step of enzyme dissociation from the complex with nicked product (Figure 5). As well as in the case of the undamaged ligand, experimental data obtained for the specific substrates did not permit the discovery of the quantity of conformations possessed by protein at the initial moment. It is conjecture, that if the protein exists in two conformational forms, the conformational transition is strongly shifted to the form which is highly active for AP- and F-substrates cleavage.

For both the wild-type protein and the truncated APE1ΔN61 lacking the N-terminal 61 amino acids we didn't register the isomerization of enzymes in the complex with the nicked product [29]. Such an isomerization stage probably exists for all three enzyme forms studied, however only the lysine-98 substitution by alanine permits the recording of this process. Our hypothesis explains why the Trp fluorescence intensities do not return to their initial values on Trp fluorescence traces of interactions of all three enzyme forms with F- and AP-substrates. Besides, this hypothesis is in agreement with the data obtained in the study [35].

The lysine-98 substitution by alanine does not have an influence on the values of forward rate constants of primary complexes formation (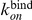) for F- or AP-substrates, as the corresponding constants for APE1 and APE1K98A barely differ (Table 2). At the same time, the reverse rate constant value of formation of primary complex (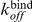) of APE1K98A with substrate F/G decreases twofold in comparison with APE1 (Table 2). Thus the stability of the complex (ES)1 of APE1K98A with F/G exceeds the stability of the corresponding APE1 complex twofold. The forward rate constant of isomerization of the primary complex with F/G (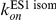) is twofold lower and the reverse constant (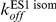) is 17-fold higher for APE1K98A than for APE1 (Table 2). Thereby in the case of interaction with F-substrate the mutation K98A leads to a 34-fold decrease in the stability of the catalytically active complex (ES)2. Moreover, this amino acid substitution leads to a 12-fold decrease in the rate constant value of hydrolysis of 5′-phosphodiester bond (*k* ^cut^) in F-substrate (Table 2). In the case of the natural AP site the mutation K98A leads to a significant increase in the values of reverse rate constant of formation of primary enzyme-substrate complex (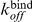), and of forward (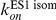) and reverse (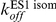) rate constants for isomerization of this complex (Table 2). Hence, the stability of (ES)1 complex of mutant protein decreases 4-fold, whereas the stability of (ES)2 complex slightly increases (1.4-fold). The values of the equilibrium dissociation constants of enzyme-product complexes are almost identical for APE1K98A (0.44 µM) and APE1 (0.48 µM [29]), indicating, that lysine-98 does not participate in the equilibrium dissociation of enzyme from the complex with the nicked product.

Our data reveal that the mutation K98A considerably obstructs the induced fit leading to the formation of the catalytically active complex (ES)2 upon interaction of the enzyme with F-containing DNA. Besides, this mutation decreases the rate of hydrolysis of 5′-phosphodiester bond in F-substrate. The Lys-98 substitution by Ala appreciably decreases the stability of the primary complex (ES)1 upon interaction of the enzyme with DNA, containing the natural AP site. Moreover, this mutation accelerates the conformational transitions in the enzyme complex with AP-substrate. APE1 is seen to interact differentially with AP site and tetrahydrofuran that is widely used as a stable analog of AP site.

### Interaction of APE1K98A with specific DHU-substrate in NIR buffer

We are of the belief (Figure 9) that at the initial moment one part of APE1K98A exists in the more active conformation E1 in relation to DHU-substrate binding. The less active form of the enzyme E2 can either form the initial enzyme–substrate complex (ES)1 or can be in slow equilibrium with the more active form E1. The enzyme in the more active form E1, rapidly generates the initial enzyme-substrate complex (ES)1 that undergoes two conformational transitions into (ES)2 and (ES)3. These stages are followed by the hydrolysis of 5′-phosphodiester bond of the substrate with the formation of enzyme complex with the reaction product (EP)1. (EP)1 then transforms slowly in (EP)2. After that the limiting process of enzyme E release from the stable complex (EP)2 occurs. In comparison with the kinetic scheme of the DHU-substrate incision by APE1 [24], in the case of APE1K98A the scheme contains an additional stage of enzyme-substrate complex isomerization.

The rate constant 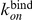 (Table 3) and the apparent forward rate constant of formation of primary complex of wtAPE1 with DHU-substrate (2.9×10^6^ M^−1^ s^−1^ [29]) are of the same order of magnitude. These constants cannot be compared more precisely. Hence, in all likelihood, the lysine-98 substitution by alanine does not have an influence on the value of the forward rate constant of primary complex formation of E1 with DHU-substrate, us in the case of F- and AP-substrates. At the same time, in the case of APE1K98A the value of 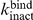 decreases 84-fold in comparison with APE1 (Table 3). The rate constant of chemical cleavage of DHU-substrate is approximately 200-fold lower for the mutant enzyme in comparison with wild-type protein (Table 3). What is more, the K98A mutation leads to an order of magnitude decrease in the rate of slow conformational conversion of enzyme-product complex. The values of rate constants of the limiting stage of enzymes release from the complexes with the nicked product (Table 3), as well as the values of equilibrium dissociation constants of enzyme-product complexes (*K*_P_^APE1K98A^ = 1.9 µM, *K*_P_^APE1^ = 1.4 µM [24]) are almost the same for both enzyme forms. Thus, Lys-98 substitution does not influence the stage of dissociation of the enzyme-product complex.

The rate constant *k* ^cut^ of DHU-substrate hydrolytic cleavage by APE1K98A in NIR buffer is approximately 23-fold lower than corresponding *k* ^cut^ for F-substrate in BER buffer (Table 2, 3). While in the case of wtAPE1 *k* ^cut^ for DHU-substrate in NIR buffer are only slightly lower than *k* ^cut^ for F-substrate and nearly twofold lower than the corresponding constant for AP-substrate in BER buffer (Table 2, 3). Hence, the substitution of lysine-98 by alanine influences the catalysis during nucleotide incision repair more than during base excision repair. In the work [25] the mutant APE1K98A also was shown to exhibit a more pronounced reduction of NIR activity compared to BER activity.

The results displayed herein clearly reveal that the substitution of lysine-98 by alanine significantly delays the activation (see Figure 9) of the less active form of the enzyme E2, as well as the hydrolysis of 5′-phosphodiester bond of DHU-substrate in the nucleotide incision repair pathway. Moreover, this amino acid substitution slows down the conformational conversion of the complex of enzyme with the product of DHU-substrate cleavage.

In conclusion, we propose that the enzymatic stages shown to be affected by K98A substitution require the proper coordination of Mg^2+^ in the metal binding site A postulated in [20]. Earlier [10] it was shown that for the efficient DHU-substrate incision by APE1, a low magnesium concentration (<2 mM) was required. On the contrary, the concentration of at least 5 mM MgCl_2_ was shown to be necessary for the efficient F-substrate incision [10]. We propose that the structure of APE1 is more labile at low magnesium concentration; therefore the protein can reach easier the conformation allowing the efficient specific interaction with such bulky lesion as DHU. High magnesium concentration stabilizes the APE1 conformation optimal for AP-substrate incision. The K98A substitution leads to the disorder of a hydrogen bonding network in the metal binding site A. The results presented here demonstrate that the substitution of lysine-98 by alanine delays the catalysis during nucleotide incision repair (0.01 mM MgCl_2_) much more than during base excision repair (5 mM MgCl_2_). Taken in tandem, these data suggest that the coordination of Mg^2+^ in the disordered metal binding site A is more efficient at a high magnesium concentration than at a low concentration. The divalent metal ion coordinated in the site A is necessary for the DHU-substrate incision as well as for the incision of AP-site, as we demonstrate that the K98A mutation influences both the DHU- and F-substrate cleavage. From these data, we propose that APE1 most probably uses the same active site to catalyze the cleavage of DHU- and AP-substrates. This correlates with data described in [36], demonstrating that APE1 appears to use the same active site to catalyze the cleavage of 3, *N* ^4^-benzetheno-dC and AP-site. Although in the study [19] the APE1 hydrophobic pocket interacting with the hydrophobic side of the abasic deoxyribose was shown to be specific for the α-anomer of the natural AP site, the protein is probably able to form the conformation, providing the proper orientation of DHU in the active site.
